# Complete genome sequence and description of *Salinispira pacifica* gen. nov., sp. nov., a novel spirochaete isolated form a hypersaline microbial mat

**DOI:** 10.1186/1944-3277-10-7

**Published:** 2015-02-09

**Authors:** Wajdi Ben Hania, Manon Joseph, Peter Schumann, Boyke Bunk, Anne Fiebig, Cathrin Spröer, Hans-Peter Klenk, Marie-Laure Fardeau, Stefan Spring

**Affiliations:** 1 Laboratoire de Microbiologie IRD, MIO, Aix Marseille Université, Marseille, France; 2 Leibniz Institute DSMZ – German Collection of Microorganisms and Cell Cultures, Braunschweig, Germany; 3 Current address: Helmholtz Centre for Infection Research, Braunschweig, Germany; 4 Current address: School of Biology, Newcastle University, Newcastle upon Tyne, UK

**Keywords:** *Spirochaetes*, Fermentative metabolism, Oxygen tolerance, Hypersaline microbial mat, Kiritimati atoll

## Abstract

During a study of the anaerobic microbial community of a lithifying hypersaline microbial mat of Lake 21 on the Kiritimati atoll (Kiribati Republic, Central Pacific) strain L21-RPul-D2^T^ was isolated. The closest phylogenetic neighbor was *Spirochaeta africana* Z-7692^T^ that shared a 16S rRNA gene sequence identity value of 90% with the novel strain and thus was only distantly related. A comprehensive polyphasic study including determination of the complete genome sequence was initiated to characterize the novel isolate.

Cells of strain L21-RPul-D2^T^ had a size of 0.2 – 0.25 × 8–9 μm, were helical, motile, stained Gram-negative and produced an orange carotenoid-like pigment. Optimal conditions for growth were 35°C, a salinity of 50 g/l NaCl and a pH around 7.0. Preferred substrates for growth were carbohydrates and a few carboxylic acids. The novel strain had an obligate fermentative metabolism and produced ethanol, acetate, lactate, hydrogen and carbon dioxide during growth on glucose. Strain L21-RPul-D2^T^ was aerotolerant, but oxygen did not stimulate growth. Major cellular fatty acids were C_14:0_, iso-C_15:0_, C_16:0_ and C_18:0_. The major polar lipids were an unidentified aminolipid, phosphatidylglycerol, an unidentified phospholipid and two unidentified glycolipids. Whole-cell hydrolysates contained L-ornithine as diagnostic diamino acid of the cell wall peptidoglycan. The complete genome sequence was determined and annotated. The genome comprised one circular chromosome with a size of 3.78 Mbp that contained 3450 protein-coding genes and 50 RNA genes, including 2 operons of ribosomal RNA genes. The DNA G + C content was determined from the genome sequence as 51.9 mol%. There were no predicted genes encoding cytochromes or enzymes responsible for the biosynthesis of respiratory lipoquinones.

Based on significant differences to the uncultured type species of the genus *Spirochaeta*, *S. plicatilis*, as well as to any other phylogenetically related cultured species it is suggested to place strain L21-RPul-D2^T^ (=DSM 27196^T^ = JCM 18663^T^) in a novel species and genus, for which the name *Salinispira pacifica* gen. nov., sp. nov. is proposed.

## Introduction

In several marine to hypersaline coastal environments cyanobacterial mats can be found that are associated with microbialites. Occasionally, the observed microbialite structures resemble stromatolites formed by thin lithifying biofilms [1]. In contrast, microbial mats covering the hypersaline, evaporitic Lake 21 on the Kiritimati atoll (Kiribati, Central Pacific) are characterized by thick reticulate microbialites that form deep below the mat surface in the dark and anoxic zone [2]. Therefore, an alkalinization of the aqueous milieu by CO_2_ assimilating photoautotrophic cyanobacteria cannot play a key role in the calcification process as suggested for most fossil and modern stromatolites [3]. Rather, it is assumed that in Lake 21 microbial mats anaerobic bacteria stimulate lithification, for instance by degradation of extracellular polymeric substances (EPS) mainly comprising the mat matrix. Disintegration of the dense and negatively charged mat matrix characterizing cyanobacterial mats could lead to the release of bound calcium ions and the reduction of steric effects inhibiting mineral precipitation [2]. In several comprehensive cultivation-independent studies using 16S ribosomal RNA genes for analyzing the composition of bacterial communities inhabiting hypersaline microbial mats it was revealed that members of the *Spirochaetes* phylum play a major role in these highly diverse ecosystems and are among the most frequently detected phylotypes [4-6]. The observed stratification of sequences affiliated to *Spirochaetes* in hypersaline mats suggests that they represent a major part of the anaerobic microbial community with a peak abundance in the suboxic zone of the mat [5,6] thereby indicating their involvement in anaerobic mineralization processes. The targeted isolation of spirochaetes was therefore a major goal in a cultivation-based survey of the microbial community of Lake 21 microbial mats [7]. In that study *Spirochaeta*-like morphotypes were frequently detected in enrichment cultures that were inoculated with anoxic mat samples and contained a pullulan derivate (Red Pullulan) as carbon source, which suggests a participation of spirochaetes in polysaccharide degradation. From such enrichments a novel strain with a *Spirochaeta*-like morphology was isolated and designated L21-Rpul-D2^T^. Phylogenetic analyses based on 16S rRNA gene sequences placed this strain within a clade of halophilic and/or alkaliphilic species within the genus *Spirochaeta*. The closest phylogenetic neighbor was the type strain of *Spirochaeta africana* sharing only a 16S rRNA gene sequence identity value of 90%, which is below the genus level according to current phylogenetic concepts [8,9]. In this study we present a detailed and comprehensive characterization of the phenotype of this strain along with the determination of the complete genome sequence. Our results suggest to place strain L21-Rpul-D2^T^ in a novel species and genus, for which the name *Salinispira pacifica* gen. nov., sp. nov. is proposed.

## Classification and features

### Strains and cultivation conditions

Strain L21-RPul-D2^T^ was isolated from an anaerobic enrichment culture inoculated with slurries of a cyanobacterial mat retrieved from the hypersaline Lake 21 on the Kiritimati atoll (Northern Line Islands, Republic of Kiribati). The location of the sampling site and details of the isolation method were described elsewhere [7].

For the preparation of media and incubation under anoxic conditions the anaerobe cultivation technique of Hungate [10] with the modifications introduced by Bryant [11] was used. The basal medium for the characterization of strain L21-RPul-D2^T^ included per liter: sea salts (50.0 g), yeast extract (2.0 g), Biotrypticase (2.0 g), L-cysteine-HCl × H_2_O (0.5 g), Balch trace element solution (10.0 ml) [12] and 1.0 ml of a 0.1% (w/v) solution of resazurin. The pH was adjusted to 7.2 with 10 M KOH solution and the medium was boiled under a stream of O_2_ free N_2_ gas and cooled to room temperature. Aliquots of 5 ml were dispensed into Hungate-type tubes, degassed under 80% N_2_ and 20% CO_2_ gas mixture, and subsequently sterilized by autoclaving at 120°C for 20 min. Before inoculation aliquots of the following sterile anoxic stock solutions were injected into the tubes containing 5 ml of medium: 0.1 ml of 10% (w/v) NaHCO_3_, 0.1 ml of 2% (w/v) Na_2_S × 9H_2_O, and 0.05 ml of 30% (w/v) MgCl_2_ × 6 H_2_O. For routine cultivation 20 mM D-glucose was added to the medium from a 1 M sterile anoxic stock solution.

For comparison the following type strains of related alkaliphilic *Spirochaeta* species were obtained from the DSMZ culture collection (Braunschweig, Germany): *S. asiatica* DSM 8901^T^, *S. africana* DSM 8902^T^, and *S. dissipatitropha* DSM 23605^T^. All of these strains were cultured according to the recommendations given in the DSMZ catalogue of strains [13] except that the DSMZ medium 1263 was used instead of DSMZ medium 700 for growing the strains DSM 8901^T^ and DSM 8902^T^.

### Phylogeny

The determination of the almost complete 16S rRNA gene sequence of strain L21-RPul-D2^T^ was already described in [7] and deposited in the GenBank/EMBL/DDBJ databases under the accession number KC665949. Phylogenetic trees based on almost complete 16S rRNA gene sequences with a minimum length of 1300 nucleotides were reconstructed using distance matrix (neighbor-joining), parsimony and maximum-likelihood programs included in the ARB package [14]. The dataset of aligned and almost complete 16S rRNA gene sequences was based on the ARB SILVA database release 111 (July 2012) [15].

**Figure 1:**
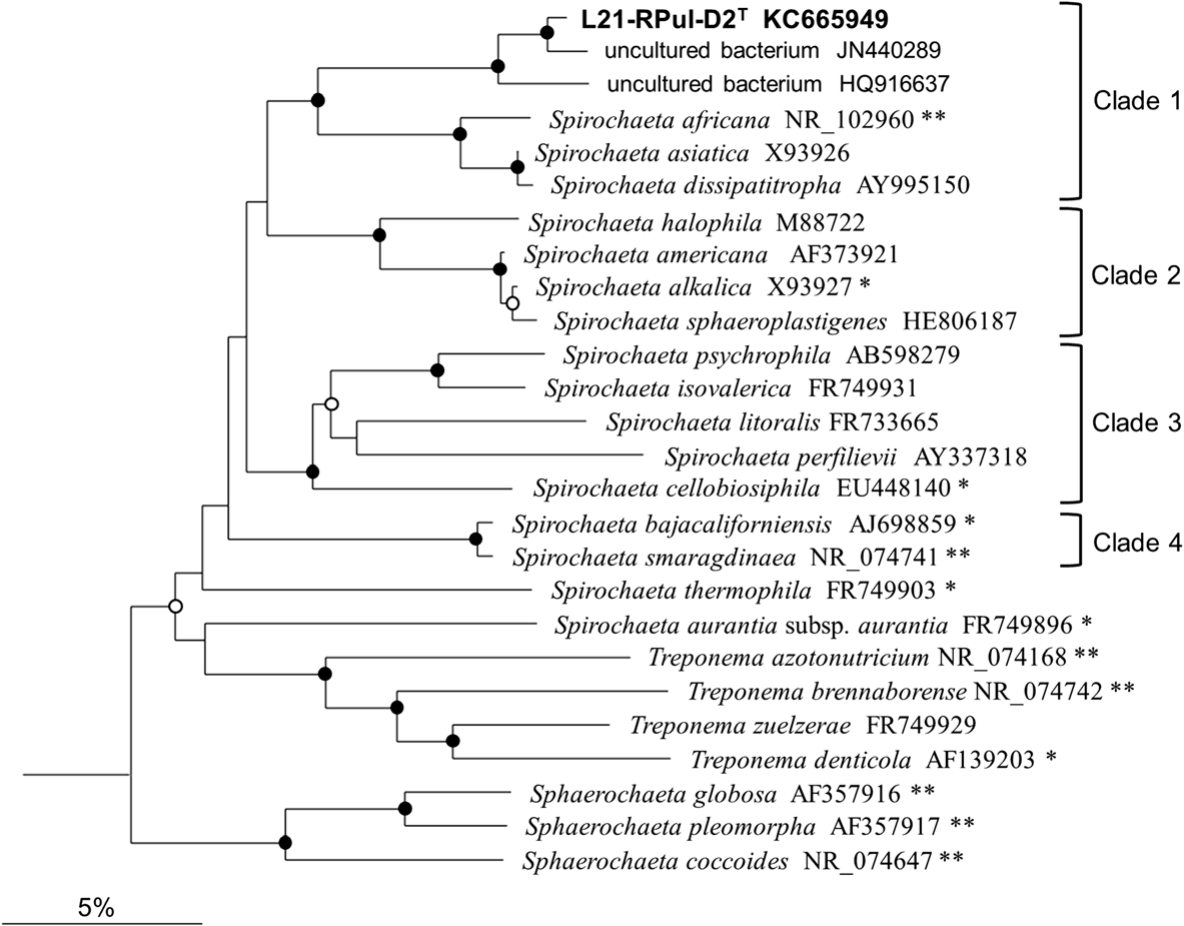
**Phylogenetic tree highlighting the position of strain L21-RPul-D2**^**T **^**within the family *Spirochaetaceae.*** The dendrogram is based on almost complete 16S rRNA gene sequences and was reconstructed with a neighbor-joining distance matrix program as implemented in the ARB package using phylogenetic distances calculated with the algorithm of Jukes and Cantor. No filter or weighting masks were used to constrain the used positions of the alignment. In addition, trees were reconstructed using the PHYLIP maximum parsimony program of ARB and the RAxML maximum likelihood program. Support of a distinct branching by bootstrap analyses is indicated by symbols. Closed circles at a distinct node indicate that bootstrap values of 80% or above (percentages of 1000 resamplings) were obtained with three different reconstruction methods, while open circles indicate that values of 80% or above were obtained with only two reconstruction methods. Stable clades of *Spirochaeta* species formed by two or more type strains are marked with brackets. The sequence of *Leptospira interrogans* (acc. no. Z12817) was used as outgroup (not shown). Type strains with genome sequencing projects registered in GOLD [20] are labeled with one asterisk, those also listed as ‘Complete and Published’ with two asterisks (see [21-23], CP002541 for *S. globosa*, CP003155 for *S. pleomorpha*, CP001841 for *Treponema azotonutricium,* and CP002696 for *T. brennaborense*). The bar represents an estimated sequence divergence of 5%.

Based on the comparative sequence analysis of 16S rRNA genes strain L21-Rpul-D2^T^ was most closely related to *Spirochaeta* species. The genus *Spirochaeta* comprises currently more than 20 species with validly published names [16]. Members of this genus are Gram-negative free-living bacteria that are widely spread in aquatic habitats, especially mud or sediments. The hallmark of spirochaetes is their slender helical cell shape combined with polar inserted periplasmic flagella conferring a spiral motility with bending and undulating movements. Despite a common morphotype free-living spirochaetes are metabolic versatile and include highly diverse phenotypes like obligately anaerobic, aerotolerant, halophilic, alkaliphilic, thermophilic and psychrophilic representatives. The observed phenotypic diversity is reflected in a large phylogenetic and genotypic divergence. The genomic DNA G + C content varies within the *Spirochaeta* from 39 – 65 mol% [17], which exceeds the variation typically found among representatives of one genus by far [18]. Accordingly, the 16S rRNA gene sequence identity values among type strains of the genus *Spirochaeta* are in the range of 99.6 to 82%, which is typically found among strains related at family or order level, but not within a genus. Several recent taxonomic studies have identified a 16S rRNA gene identity value of 95% or above for strains belonging to one genus [8,9,19]. This indicates that the genus *Spirochaeta* is currently only loosely defined on the molecular level and comprises several misclassified species. In reconstructed phylogenetic trees based on 16S rRNA gene sequences strain L21-Rpul-D2^T^ was affiliated with high bootstrap support to a clade of *Spirochaeta* species isolated from alkaline or saline environments (Figure 1). A BLAST search against the nr/nt database including 16S rRNA gene sequences of non-cultured bacteria resulted in several hits with identity values above 97%. The best matches were found with two clone sequences retrieved from a hypersaline microbial mat (JN449894 and JN440289) indicating a preference of strains related to L21-RPul-D2^T^ for microbial mats. Another more distantly related cloned 16S rRNA gene sequence (HQ916637) with an identity value of 97% was retrieved from a terrestrial mud volcano in Taiwan. Based on 16S rRNA sequence identity values the closest related type strains were *S. africana* DSM 8902^T^ (90%), *S. asiatica* Z-7591^T^ (89%) and *S. dissipatitropha* ASpC2^T^ (89%), which form together with strain L21-RPul-D2^T^ a common lineage supported by high bootstrap values above 80%. Five further stable clades could be identified by bootstrap analyses, of which two were so far only represented by one species with a validly published name (Figure 1).

### Morphology and physiology

#### Shape and pigmentation

**Figure 2:**
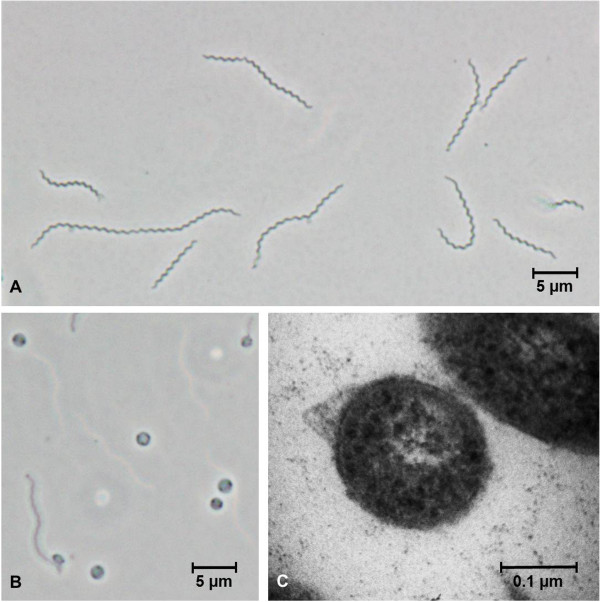
**Morphological characteristics of cells.** Phase contrast micrographs of strain L21-RPul-D2^T^ showing the typical helical shape of exponentially grown cells **(A)** and spherical bodies during late stationary phase **(B)**. Electron micrograph of a transversal cross-section through the middle of a cell showing a bulging outer sheath encasing two putative periplasmic flagella **(C)**.

The Gram reaction of cells of strain L21-RPul-D2^T^ was determined with air-dried smears of liquid cultures that were fixed with methanol and stained with DIFCO kit reagents. For electron microscopy, exponentially grown cells were negatively stained with 1% sodium phosphotungstic acid (pH 7.2). Whole cells and cross-sections were observed with a Zeiss EM 912 electron microscope at an accelerating voltage of 75 kV. The cross-sections were obtained by fixation of cells with 10% glutaraldehyde and 0.1 M cacodylate buffer, a post fixation staining with 2% osmium tetroxide and subsequent inclusion in epoxy resin (EMbed 812). The presence of spores was analyzed by phase contrast microscopic observations of young and old cultures and pasteurization tests, performed at 80, 90 and 100°C for 10 and 20 min. Cells of strain L21-RPul-D2^T^ stained Gram-negative, had a slender helical shape and exhibited a rotating, undulatory motility typical of spirochaetes. In exponential growth phase the size of cells was 0.2 - 0.25 μm in width and 8–9 μm in length with an approximate wavelength of 1 μm (Figure 2A). Sometimes cells up to 25 μm in length were observed. Coccoid bodies resembling spheroplasts appeared in stationary phase cultures and had a diameter ranging from 1.5 to 2.8 μm (Figure 2B). The ultrastructure of cells is characterized by two periplasmic flagella that overlap in the middle of the cell (Figure 2C).

Pigments were extracted for spectroscopic analyses from strain L21-RPul-D2^T^ and related type strains of *Spirochaeta* with a mixture of acetone/methanol (7:2 v/v) as described previously [24]. Spectra were recorded with a Thermo BioMate 6 UV–VIS split beam spectrophotometer. Pigments were formed under anaerobic and semiaerobic incubation conditions and gave cells a yellow to light orange color. Absorption spectra of the pigments extracted with acetone/methanol were characteristic for carotenoids with maxima at 439 and 468 nm and a shoulder at 487 nm. Carotenoid-like pigments could be also extracted from cell pellets of the type strains of *S. africana* and *S. dissipatitropha*, but not of *S. asiatica*.

#### Growth conditions

The pH, temperature and NaCl concentration ranges for growth were determined using basal medium supplemented with 20 mM D-glucose. Different pH values (5 to 9) of the medium were adjusted in increments of around 0.5 by injecting aliquots of anoxic stock solutions of 100 mM HCl (acidic pH values), 10% (w/v) NaHCO_3_ or Na_2_CO_3_ (basic pH values) in Hungate-type tubes containing 5 ml of medium. Water baths were used for incubating bacterial cultures from 15 to 55°C. NaCl requirement was determined by directly weighing NaCl in Hungate-type tubes before dispensing modified basal medium containing per liter: Na_2_SO_4_ (5.70 g), KCl (1.00 g), KBr (0.04 g), yeast extract (2.00 g), Biotrypticase (2.00 g), L-cysteine-HCl (0.50 g), NaHCO_3_ (0.30 g), resazurin (1.00 mg) and 10.00 ml of Balch trace element solution. Strain L21-RPul-D2^T^ was moderately halophilic and grew optimally at a salinity of 5% (w/v). It required at least 2% (w/v) NaCl for growth and tolerated salinities up to 15% (w/v), which is the highest known salt tolerance of any known member of the free-living spirochaetes and grew at temperatures ranging from 20 to 45°C, with an optimum at 35°C. The pH range for growth was 6.5–8.4, with an optimum at pH 6.9-7.0.

#### Substrate utilization

Substrates (D-glucose, D-ribose, sucrose, D-fructose, D-xylose, D-arabinose, lactose, D-maltose, D-mannose, D-melibiose, D-cellobiose, D-trehalose, glycerol, ethanol, methanol, formate, acetate, pyruvate, fumarate, DL-lactate, succinate, DL-malate, citrate, butyrate, propionate) were tested at a final concentration of 20 mM in glucose-free basal medium. Casamino acids, peptone, starch and pullulan were tested at a final concentration of 2 g l^-1^ and 80% H_2_ and 20% CO_2_ gas mixture with and without acetate (2 mM) was tested under 2 bars of overpressure. To test for electron acceptors, sodium thiosulfate (20 mM), sodium sulfate (20 mM), sodium sulfite (2 mM), elemental sulfur (10 g l^-1^), sodium nitrate (20 mM), or sodium nitrite (2 mM) were added to the medium. Cultures were subcultured at least twice under the same experimental conditions before determination of growth rates. H_2_S production was determined photometrically as colloidal CuS according to Cord-Ruwisch [25]. End-products of metabolism were measured by high pressure liquid chromatography (HPLC) after 2 days of incubation at 35°C [26]. Strain L21-RPul-D2^T^ had a strictly fermentative-type of metabolism and required yeast extract or Trypticase peptone for growth, but no vitamins. It was saccharolytic and used pullulan, starch, N-acetylglucosamine, D-fructose, D-glucose, D-maltose, D-mannose, and D-trehalose for growth, but not peptides or amino acids. In addition the carboxylic acids fumarate and pyruvate were utilized as electron donors. No positive growth response was obtained with chitin, arabinose, cellobiose, D-galactose, lactose, melibiose, D-ribose, sucrose, D-xylose, acetate, butyrate, casamino acids, citrate, formate, lactate, malate, propionate, succinate, ethanol, glycerol, D-mannitol, methanol, and H_2_/CO_2_ (8:2 v/v). The end-products resulting from D-glucose fermentation were acetate, lactate, ethanol, CO_2_ and H_2_. Sulfate, sulfite, elemental sulfur, nitrate and nitrite were not used as terminal electron acceptors. Thiosulfate and nitrate were not reduced during anaerobic growth. Despite a negative reaction of cells in tests for oxidase and catalase activity, growth did not depend on prereduced media. Oxygen concentrations of up to 10% were tolerated in the headspace gas atmosphere of statically incubated cultures. During semiaerobic cultivation (5 – 10% O_2_ in the headspace gas atmosphere) the growth yield did not increase compared to anaerobic incubation and the pattern of fermentation products did not change significantly, hence oxygen was not used as electron acceptor for respiratory metabolism.

#### Resistance to antibiotics

Susceptibility to antibiotics was tested by adding sterile anoxic stock solutions of antibiotics to the complete medium prior to inoculation. Rifampicin and chloramphenicol were dissolved in methanol, while stock solutions of all other antibiotic compounds were prepared in distilled water. Final concentrations of the respective antibiotics in cultivation media were 10, 100 and 1000 mg l^-1^ for ampicillin, carbenicillin, D-cycloserin, gentamicin, kanamycin A and penicillin G; 10, 100 and 500 mg l^-1^ for tetracycline; 10 and 100 mg l^-1^ for rifampicin; 20 and 200 mg l^-1^ for chloramphenicol. Strain L21-RPul-D2^T^ was resistant to the antibiotics rifampicin (10–100 mg l^-1^) and kanamycin A (10–1000 mg l^-1^), but susceptible to ampicillin (1000 mg l^-1^), carbenicillin (1000 mg l^-1^), penicillin G (1000 mg l^-1^), D-cycloserin (1000 mg l^-1^), chloramphenicol (20 mg l^-1^), gentamicin (1000 mg l^-1^), and tetracycline (500 mg l^-1^). A summary of the classification and general features of strain L21-RPul-D2^T^ is presented in Table 1 and a list of diagnostic traits allowing differentiation from related type strains is given in Additional file 1.

**Table 1:** **Classification and general features of *Salinispira pacifica *** **L21-Rpul-D2**^**T **^**in accordance with the MIGS recommendations **[27]** published by the Genomic Standards Consortium **[28]

**MIGS ID**	**Property**	**Term**	**Evidence code**
	Current classification	Domain *Bacteria*	TAS [29]
		Phylum *Spirochaetes*	TAS [30]
		Class “*Spirochaetia”*	TAS [30]
		Order *Spirochaetales*	TAS [31]
		Family *Spirochaetaceae*	TAS [31]
		Genus *Salinispira*	IDA
		Species *Salinispira pacifica SRubellilLLeisingOwenweeksiahongkongensis*	IDA
		Type strain L21-RPul-D2^T^	IDA
	Gram stain	negative	IDA
	Cell shape	helical-shaped	IDA
	Motility	motile	IDA
	Sporulation	non-sporulating	IDA
	Temperature range	20-45°C	IDA
	Optimum temperature	35°C	IDA
	Salinity range	2-15% (NaCl)	IDA
	Optimum Salinity	5% (NaCl)	IDA
MIGS-22	Oxygen requirement	anaerobic, oxygen-tolerant	IDA
	Carbon source	carbohydrates, carboxylic acids	IDA
	Energy metabolism	chemoheterotroph, fermentative	IDA
MIGS-6	Habitat	hypersaline microbial mat	TAS [7]
MIGS-15	Biotic relationship	free-living	TAS [7]
MIGS-14	Pathogenicity	none	NAS
	Biosafety level	1	NAS
MIGS-23.1	Isolation	anoxic mat sample	TAS [7]
MIGS-4	Geographic location	Lake 21, Kiritimati, Kiribati (Central Pacific)	TAS [7]
MIGS-5	Sample collection time	March 2011	TAS [7]
MIGS-4.1	Latitude	1°57.87′ N	TAS [7]
MIGS-4.2	Longitude	157°20.04′ W	TAS [7]
MIGS-4.3	Depth	0.1 m below surface	TAS [7]

Evidence codes - IDA: Inferred from Direct Assay; TAS: Traceable Author Statement (i.e., a direct report exists in the literature); NAS: Non-traceable Author Statement (i.e., not directly observed for the living, isolated sample, but based on a generally accepted property for the species, or anecdotal evidence). Evidence codes are from of the Gene Ontology project [34].

### Chemotaxonomy

The cellular fatty acid pattern of strain L21-RPul-D2^T^ was determined from cells grown to early stationary phase in TYG medium [7]. For comparison additional cellular fatty acid patterns were determined from related type strains cultured in DSMZ medium 1263 under the conditions given in the DSMZ catalogue of strains [13]. The preparation and extraction of fatty acid methyl esters from biomass and their subsequent separation and identification by gas chromatography was done as described elsewhere [35]. Polar lipid analyses were carried out by the DSMZ Identification Service according to the published protocols [36]. Diagnostic diamino acids of the cell wall peptidoglycan were detected in hydrolysates (4 N HCl, 100°C, 16 h) of whole cells by using GC/MS as described by Schumann [37]. The cellular fatty acid composition of strain L21-RPul-D2^T^ in comparison with the profiles of phylogenetically related *Spirochaeta* strains is shown in Additional file 2. The major cellulary fatty acids (>5% of the total abundance) of the novel isolate were C_14:0_, C_16:0_, iso-C_15:0_, and C_18:0_. Unique characteristics of strain L21-RPul-D2^T^ compared to all related *Spirochaeta* strains were a predominance of the fatty acids C_18:0_ (9.7%) and iso-C_15:0_ (11.7%) combined with the presence of the branched fatty acid ante-C_15:0_. The determined polar lipid pattern revealed major amounts of phosphatidylglycerol, an unidentified aminolipid, an unidentified phospholipid and two distinct glycolipids (Additional file 3). The cell wall peptidoglycan contained L-ornithine as diagnostic diamino acid (type A1β according to the classification of Schleifer and Kandler [38]), which is a characteristic of the family *Spirochaetaceae*[30].

## Genome sequencing and annotation

### Genome project history

The genome of strain L21-RPul-D2^T^ was sequenced within the DFG funded project “Microbial control of mineralization processes in non-marine biofilms and microbial mats”. The strain was chosen for genome sequencing according to its low 16S rRNA gene sequence identity value to related type strains.

Project information is available from the Genomes OnLine Database [20] and the complete genome sequence can be found at GenBank. A summary of the project information is shown in Table 2.

**Table 2:** Genome sequencing project information

**MIGS ID**	**Property**	**Term**
MIGS-13	Source material identifier	DSM 27196^T^
MIGS-31	Finishing quality	complete
MIGS-28	Libraries used	Three genomic libraries: PacBio SMRTbell™ library (> 10 kb) for draft assembly; Illumina PE library (350 bp insert size) and 454 PE library (3 kb insert size) for error correction
MIGS-29	Sequencing platforms	PacBio RSII, Illumina GA IIx, 454
MIGS-31.2	Fold coverage	365 (PacBio)
MIGS-30	Assemblers	HGAP 2 (SMRTPortal 2.1.0), BWA
MIGS-32	Gene calling method	RAST
	Locus Tag	L21SP2
	GenBank ID	CP006939.1
	GenBank Date of Release	February 14, 2014
	GOLD ID	Gp0075701
	NCBI project ID	227794
	Project relevance	Environmental, biodiversity

### Growth conditions and DNA isolation

A culture of strain L21-RPul-D2^T^ was grown anaerobically in TYG medium [7] at 35°C. Genomic DNA was isolated using Jetflex Genomic DNA Purification Kit (GENOMED 600100) following the standard protocol provided by the manufacturer but modified by an incubation time of 60 min, incubation on ice overnight on a shaker, the use of additional 50 μl proteinase K (21 mg ml^-1^), and the addition of 200 μl protein precipitation buffer. DNA is available from the Leibniz Institute DSMZ through the DNA Network [39].

### Genome sequencing and assembly

The genome was sequenced using a combination of three genomic libraries (Table 2). SMRTbell™ template library was prepared according to the instructions from Pacific Biosciences, Menlo Park, CA, USA, following the Procedure & Checklist Greater than 10 kb Template Preparation and Sequencing. Briefly, for preparation of 10 kb libraries ~10 μg genomic DNA was end-repaired and ligated overnight to hairpin adapters applying components from the DNA/Polymerase Binding Kit P4 from Pacific Biosciences, Menlo Park, CA, USA. Reactions were carried out according to the manufacturer’s instructions. SMRTbell™ template was exonuclease treated for removal of incompletely formed reaction products. Conditions for annealing of sequencing primers and binding of polymerase to purified SMRTbell™ template were assessed with the Calculator in RS Remote, Pacific Biosciences, Menlo Park, CA, USA. SMRT sequencing was carried out on the PacBio RSII (Pacific Biosciences, Menlo Park, CA, USA) taking one 120-minutes movie for each SMRT cell. In total 5 SMRT cells were run. Illumina sequencing was performed on a GA IIx platform with 150 cycles. The paired-end library contained inserts of an average insert size of 350 bp and delivered 3.0 million reads. A second Illumina run was performed on a Miseq platform to gain a higher sequencing depth. To achieve longer reads, the MiSeq library was sequenced in one direction for 300 cycles, providing another 2.0 million reads. 454 paired-end jumping library of a 3 kb insert library was performed on a 1/8 lane. Pyrosequencing resulted in 92,601 reads with an average read length of 371 bp assembled in Newbler (Roche Diagnostics).

A draft long read genome assembly named L21-Pul-V2-SP2_HGAP_5SC_std was created using the “RS_HGAP_Assembly.1” protocol included in SMRTPortal version 2.1.0 including all 5 SMRT cells. Standard parameters were applied including: Assembly – Preassembly: Compute Minimum Seed Read Length true, Allow Partial Alignments true, Trim FASTQ Output true, --- Celera Assembler v1 Genome Size (Bp) 5000000, Target Coverage 15, Overlapper Error Rate 0.06, Overlapper Min Length 40, Overlapper K-mer 14. Thus, one final contig could be obtained, which was trimmed, circularized and adjusted to *dnaA* as first gene. A total coverage of 365× has been calculated within the long read assembly process. Quality check of the final consensus sequences regarding overall coverage as well as SNPs was performed using IGV [40] after mapping of Illumina and 454 short read data onto the draft genome using BWA [41].

### Genome annotation

Genome annotation and analysis were done using the RAST server [42]. Additional gene prediction analysis and functional annotation was performed within the Integrated Microbial Genomes - Expert Review (IMG-ER) platform [32].

### Genome properties

**Figure 3:**
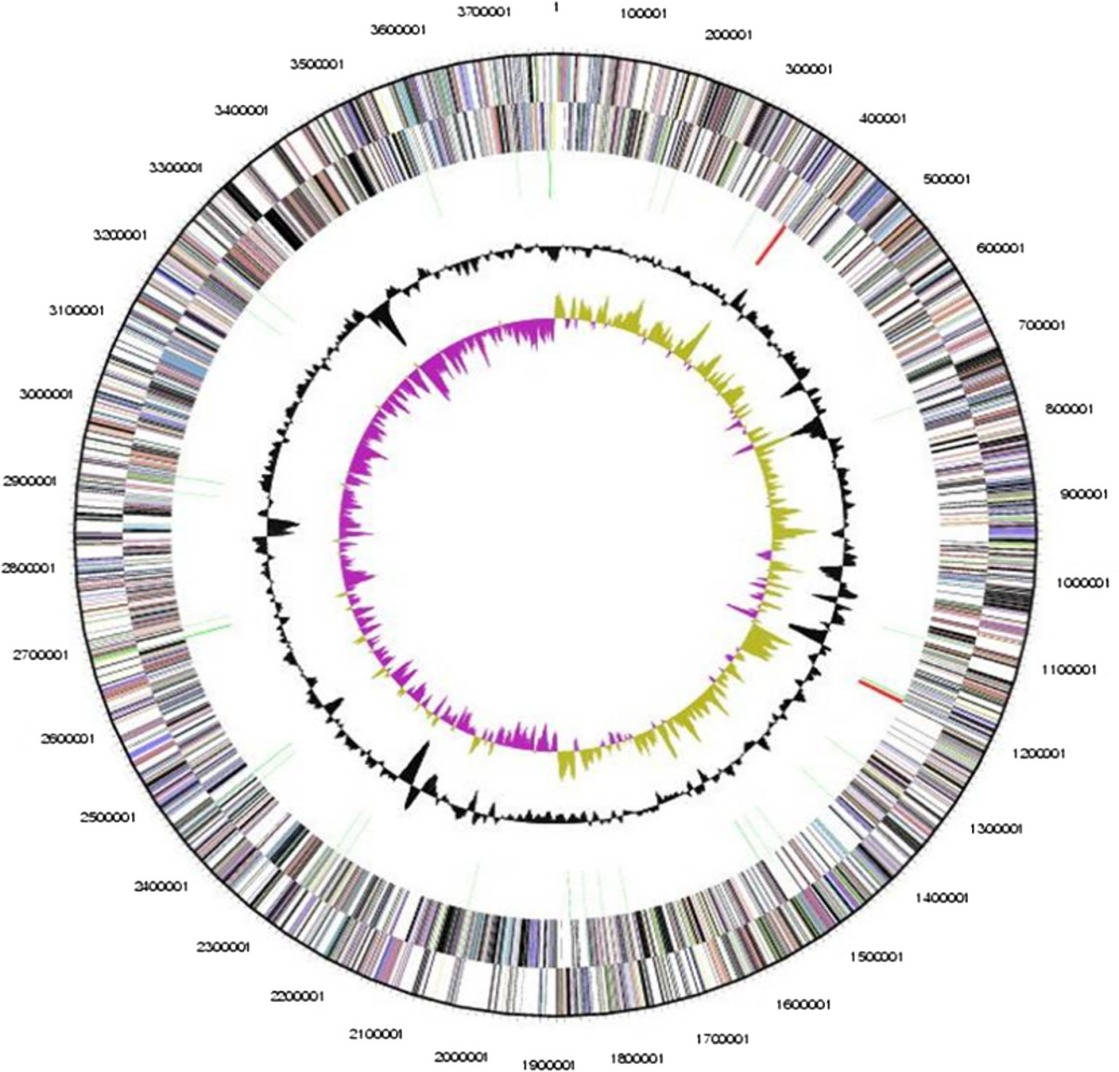
**Graphical map of the chromosome.** From outside to the center: Genes on forward strand (colored by COG categories), Genes on reverse strand (colored by COG categories), RNA genes (tRNAs green, rRNAs red, other RNAs black), GC content (black), GC skew (purple/olive).

The genome of strain L21-RPul-D2^T^ is represented by one circular chromosome that has a total length of 3,782,798 bp and a G + C content of 51.86 mol%. The genome sequence has been deposited in GenBank nucleotide database under the accession number CP006939.1. The genome statistics are provided in Table 3 and Figure 3. Of the 3,500 genes predicted, 3,450 were identified protein-coding genes and 50 RNAs. Around half of the protein-coding genes were assigned a putative function (56.9%) while the remaining ones were annotated as hypothetical proteins. The distribution of genes into COGs functional categories is presented in Table 4.

**Table 3:** Genome statistics

**Attribute**	**Value**	% of Total*
Genome Size (bp)	3,782,798	100.00
DNA coding (bp)	3,435,593	90.82
DNA G + C (bp)	1,961,772	51.86
DNA scaffolds	1	100.00
Total genes	3500	100.00
Protein coding genes	3450	98.57
RNA genes	50	1.43
Pseudo genes	53	1.51
Genes with function prediction	1990	56.86
Genes in paralog clusters	2366	67.60
Genes assigned to COGs	1914	54.69
Genes with Pfam domains	2487	71.06
Genes with signal peptides	146	4.17
Genes with transmembrane helices	857	24.49
CRISPR repeats	0	

*The total is based on either the size of the genome in base pairs or the total number of protein coding genes in the annotated genome.

**Table 4:** Number of genes associated with general COG functional categories

**Code**	**Value**	% of total*	**Description**
J	137	6.44	Translation, ribosomal structure and biogenesis
A	0	0.00	RNA processing and modification
K	124	5.83	Transcription
L	119	5.59	Replication, recombination and repair
B	1	0.05	Chromatin structure and dynamics
D	20	0.94	Cell cycle control, mitosis and meiosis
V	41	1.93	Defense mechanisms
T	156	7.33	Signal transduction mechanisms
M	97	4.56	Cell wall/membrane biogenesis
N	74	3.48	Cell motility
U	42	1.97	Intracellular trafficking and secretion
O	81	3.81	Posttranslational modification, protein turnover, chaperones
C	135	6.35	Energy production and conversion
G	179	8.42	Carbohydrate transport and metabolism
E	196	9.21	Amino acid transport and metabolism
F	65	3.06	Nucleotide transport and metabolism
H	72	3.39	Coenzyme transport and metabolism
I	71	3.34	Lipid transport and metabolism
P	141	6.63	Inorganic ion transport and metabolism
Q	24	1.13	Secondary metabolites biosynthesis, transport and catabolism
R	234	11.00	General function prediction only
S	118	5.55	Function unknown
-	1586	45.31	Not in COGs

*The total is based on the total number of protein-coding genes in the genome.

### Comparative genomics

To date genome sequences of the following type strains of *Spirochaeta* species are available in public databases (accession numbers in parentheses): *S. africana* DSM 8902^T^ (CP003282) [21], *S. alkalica* DSM 8900^T^ (AREC01000000), *S. bajacaliforniense* DSM 16054^T^ (ARJU01000000), *S. cellobiosiphila* DSM 17781^T^ (AUFW01000000), *S. smaragdinae* DSM 11293^T^ (CP002116) [22] and *S. thermophila* DSM 6578^T^ (CP002903). The percentage of conserved genes among genomes was determined using the phylogenetic profiler function of the IMG-ER system [32] with default adjustments, except that the minimal percent identity for homologous genes was set to 40%. Values of percentages of conserved genes were calculated according to the POCP formula proposed by Qin et al. [33]. The shared gene content among the novel strain L21-RPul-D2^T^ and genome-sequenced *Spirochaeta* species is shown in Table 5 along with corresponding 16S rRNA gene identity values.

**Table 5:** **Shared content of conserved genes among the genomes of *Salinispira pacifica *** **strain L21-RPul-D2**^**T **^**and related type strains of *Spirochaeta***

**Type strain**	**1**	**2**	**3**	**4**	**5**	**6**	**7**
**1.***Salinispira pacifica*	-	90	88	88	86	84	87
**2.***S. africana*	35	-	90	88	87	87	88
**3.***S. alkalica*	32	40	-	88	88	86	87
**4.***S. smaragdinae*	26	28	31	-	87	87	99
**5.***S. cellobiosiphila*	27	28	27	28	-	86	87
**6.***S. thermophila*	30	34	34	30	28	-	86
**7.***S. bajacaliforniensis*	26	28	31	84	29	30	-

Upper right triangle - 16S rRNA gene identity values. Lower left triangle - percentage of conserved genes among genomes. Accession numbers of used genomes: 1, CP006939; 2, CP003282; 3, AREC01000000; 4, CP002116; 5, AUFW01000000; 6, CP002903; 7, ARJU01000000.

Values of the shared gene content were in the range from 26 to 84% and 16S rRNA gene identity values varied between 84 and 99%, which indicates an extensive genetic heterogeneity within this monophyletic group. A recent study proposed to use the percentage of conserved proteins (POCP) among two genomes for genus demarcation [33]. According to this proposal strains having POCP values below 50% and 16S rRNA gene sequence identity values below 93% should belong to different genera. Consequently, only the two species *S. smaragdinae* and *S. bajacaliforniense* would belong to the same genus by having a 16S rRNA gene identity value of 99% and sharing 85% of their gene content, all other species and the novel strain L21-RPul-D2^T^ would belong to different genera. This view is corroborated by some recently published whole genome sequence-based phylogenies containing a significant number of genome-sequenced representatives of the *Spirochaetaceae*[23,43].

### Energy metabolism

Strain L21-RPul-D2^T^ used sugars and some carboxylic acids as substrates for fermentative growth, whereas peptides or amino acids were not utilized. Monosaccharides are transported into the cell via group translocation using the phosphotransferase system (PTS). Several genes encoding phosphocarrier proteins (L21SP2_2173 and L21SP2_3294), enzyme I (L21SP2_1343 and L21SP2_3295), and enzyme II (L21SP2_0100, L21SP2_0865, L21SP2_3408, and L21SP2_3409) of a putative PTS transport mechanism were detected. In addition, genes encoding a TRAP-type dicarboxylate transporter (L21SP2_1277 and L21SP2_1278) were present. The assimilation of peptides, which had a stimulating effect on growth, is probably catalyzed by transporters of the ABC-type (e.g., L21SP2_0701 - 0706). Presence of genes encoding both subunits of the key enzyme 6-phosphofructokinase (pyrophosphate-dependent) (L21SP2_0225 and L21SP2_2454) indicates that glucose is metabolized to pyruvate through the Embden-Meyerhof-Parnas (EMP) pathway in strain L21-RPul-D2^T^. According to the genome sequence several alternative reactions are possible for the further oxidation of pyruvate to acetyl-CoA. A pyruvate dehydrogenase complex encoded by the genes L21SP2_2176 - 2179 catalyzes the oxidative decarboxylation of pyruvate with concomitant release of CO_2_ and NADH. The pyruvate dehydrogenase complex is typically found in aerobic bacteria and its presence in obligate fermentative bacteria is unusual, but maybe explained by the aerotolerant lifestyle of this strain. In contrast to the pyruvate dehydrogenase complex, both alternative enzymes found in L21-Rpul-D2^T^ are very sensitive to oxygen and operate only under anoxic conditions. The enzyme most frequently found in anaerobic bacteria for pyruvate oxidation is pyruvate:ferredoxin oxidoreductase, which is also present in L21-Rpul-D2^T^ (L21SP2_2933) and releases reduced ferredoxin instead of NADH. The third route of pyruvate oxidation is catalyzed by pyruvate:formate lyase, which produces formate and acetyl-CoA and is typically found in Gram-negative facultatively anaerobic bacteria performing a mixed acid fermentation. In L21-RPul-D2^T^ this enzyme is encoded at L21SP2_2006. The intermediate metabolite acetyl-CoA can be further oxidized to CO_2_ via the citric acid cycle, which appears to be fully functional in this strain, or reduced to ethanol by a combined acetaldehyde and alcohol dehydrogenase, both encoded by one gene at L21SP2_0358. No genes for the synthesis of respiratory lipoquinones, soluble cytochromes or membrane-bound terminal oxidases were present, which indicates that substrate-level phosphorylation is the main mechanism for the generation of ATP under anaerobic conditions. Consequently, the regeneration of NAD^+^ and oxidized ferredoxin has to be achieved by fermentative reactions, for example by reduction of pyruvate to lactate by a D-lactate dehydrogenase encoded at L21SP2_2897. Furthermore, several genes for distinct [FeFe] hydrogenases were present (e.g., L21SP2_0276, L21SP2_0545). Cytoplasmic iron-only hydrogenases are known to be involved in the regeneration of reduced ferredoxin and the production of hydrogen in some fermentative bacteria [44].

On the other hand, genes for several membrane-bound enzyme complexes were detected that could be involved in the generation or utilization of a chemiosmotic gradient without participation of an electron transport chain. For example it was found that in some fermenting thermophilic archaea a chemiosmotic gradient is generated by the oxidation of reduced ferredoxins at membrane-bound energy-coupling hydrogenases [45,46]. In strain L21-Rpul-D2^T^ a RNF-like electron transport complex encoded at L21SP2_0447 – 0452 could catalyze the oxidation of ferredoxin with NAD^+^, which has been shown to be coupled to translocation of protons or sodium ions in several anaerobic prokaryotes [47]. In addition, a multimeric complex with similarity to a sodium translocating NADH:quinone oxidoreductase (L21SP2_2184 – 2188) and a multicomponent sodium/proton antiporter (L21SP2_1997–2003) could be detected. A V-type ATP synthase is encoded by the genes L21SP2_1878–1884. This enzyme complex could function as an ATP driven ion pump as in *Streptococcus faecalis*[48] or in the utilization of a proton gradient for the synthesis of ATP as in *Thermus thermophilus*[49]*,* thereby enabling an alternative to substrate-level phosphorylation.

### Oxidative stress and carotenoid synthesis

Strain L21-RPul-D2^T^ was isolated from the subsurface layers of a cyanobacterial mat that can be exposed to changing concentrations of oxygen due to the photosynthetic activity of cyanobacteria in the light. Like several other spirochaetes strain L21-RPul-D2^T^ is aerotolerant and can grow fermentatively in the presence of oxygen, which may represent an important feature for the persistence of spirochaetes in cyanobacterial mats. Oxygen in cells of anaerobic microorganisms can have harmful effects by the formation of reactive oxygen species (ROS) at the active site of certain enzymes containing flavins [50]. To prevent oxidative stress by ROS, anaerobic bacteria have to keep intracellular concentrations of oxygen and its partially reduced derivates low. Although, it was observed that strain L21-RPul-D2^T^ actively reduces oxygen-containing cultivation media during growth, no genes encoding a putative terminal oxidase or catalase could be detected in the genome sequence, which represent the main oxygen removal mechanisms of anaerobic respiratory bacteria, e.g. sulfate reducers [51]. In contrast, most aerotolerant fermentative bacteria use a NADH oxidase system for reduction of oxygen. Detoxification with NADH can be catalyzed by a single enzyme as in *Brachyspira hyodysenteriae*[52] or by a cascade of reactions including the electron carrier proteins rubredoxin and rubrerythrin [53]. Although, no NADH oxidase gene was annotated by the RAST server two candidate genes encoding putative NADH oxidase domains (L21SP2_0477 and L21SP2_0797) were detected in the genome sequence of L21-RPul-D2^T^ by performing a BLASTP search with the NADH oxidase of *B. hyodysenteriae* (Q59917). However, at least one of both enzymes probably acts as a coenzyme A disulfide reductase that is a highly similar orthologue of NADH oxidase. Based on the absence of genes encoding glutathione oxidoreductase or enzymes for the synthesis of glutathione it can be deduced that in strain L21-RPul-D2^T^ reduced coenzyme A represents the main low-molecular-weight thiol redox buffer. It has been revealed that in *Borrelia burgdorferi* reduced coenzyme A is oxidized by H_2_O_2_ and reduced again by coenzyme A reductase at the expense of NADH [54] thereby protecting cells against oxidative stress. In addition, alternative systems for detoxification of oxygen based on rubrerythrin (L21SP2_2848) and rubredoxins (L21SP2_1281, L21SP2_2197 and L21SP2_2972) seems to be active in strain L21-RPul-D2^T^, although no genes encoding a putative NADH:rubredoxin oxidoreductase were detected in the genome sequence. Some of the endogenous formed H_2_O_2_ may be reduced to water by the alkyl hydroperoxide reductase AhpCF (L21SP2_3374, L21SP2_3375) that also uses organic hydroperoxides as substrates [55]. For removal of the highly reactive superoxide anion a manganese superoxide dismutase (L21SP2_1673) or a superoxide reductase (L21SP2_1734) could be used.

Independent of the enzymatic reduction of ROS at the expense of NAD(P)H as reductant carotenoids seem to be used by certain spirochaetes to protect cellular lipids against oxidative stress. All aerotolerant species in the clade of halophilic/alkaliphilic spirochaetes represented by L21-RPul-D2^T^ produce carotenoids, whereas the strictly anaerobic species *S. asiatica* is unpigmented (Additional file 1). Furthermore, it was observed that in some spirochaetes the production of carotenoids is induced only in the presence of oxygen [30,56]. Hydroxylated derivates of β-carotene like zeaxanthin were shown to have a high antioxidant effect by scavenging singlet oxygen [57], and thus are probably used by strain L21-RPul-D2^T^ and related *Spirochaeta* species for the protection of their cell membranes against oxidative stress. The biosynthesis of carotenoids in L21-RPul-D2^T^ depends on a phytoene synthase (L21SP2_2164) and phytoene desaturase (L21SP2_2163) that transform the precursor geranygeranyl diphosphate in lycopene, which is further converted to β-carotene by a lycopene cyclase (L21SP2_0393). Zeaxanthin could then be synthesized by a putative beta-carotene hydroxylase (L21SP2_3046).

## Conclusions

The type of the genus *Spirochaeta* is represented by a description of a distinct morphotype occurring in sulfidic freshwater or marine mud that was designated *S. plicatilis*. Characteristics of Gram-negative staining cells identified as *S. plicatilis* are a helical shape, a size of 50–250 μm in length and 0.5–0.75 μm in width and a bundle of periplasmic flagella. Long filaments may consist of multicellular chains of cells [30,58]. The conspicuous morphology of *S. plicatilis*, *i.e.* Gram-negative staining helical cells with periplasmic flagella, which was used for the definition of the genus *Spirochaeta* later turned out to be a characteristic of the whole phylum *Spirochaetes*[30] and thus is of limited value for the definition of a genus. No cultured type strain or 16S rRNA gene sequence of *S. plicatilis* is available as reference for comparative studies with other strains of this genus, thereby preventing an exact placement of this species within the phylum *Spirochaetes*. However, the morphological description of *S. plicatilis* does not fit to strain L21-RPul-D2^T^ or any other cultured species of the genus *Spirochaeta*, which do not form filaments of 50–250 μm in length, have a smaller cell width and only two periplasmic flagella [30]. Within the phylum *Spirochaetes* distinctive morphological traits seem to be conserved at genus or family level, so that for example strains that exclusively grow in the form of non motile spherical cells are found only within the genus *Sphaerochaeta* or cells with hooked ends are typical for *Leptospira*. We conclude, therefore, that all *Spirochaeta* species currently available in pure culture are most likely misclassified due to significant morphological differences to the type of this genus. Therefore, it is proposed to rename species of the genus *Spirochaeta* according to recent taxonomic concepts based on molecular methods. As outlined above, the large phenotypic and phylogenetic divergence to any cultured described strain would also suggest placing strain L21-Rpul-D2^T^ in a novel species and genus within the family *Spirochaetaceae*. The novel genus can be clearly distinguished from the most closely related species *S. africana*, *S. asiatica* and *S. dissipatitropha*, which are alkaliphilic. According to our taxonomic concept *S. asiatica* and *S. dissipatitropha* likely represent a separate genus that could be in turn distinguished from *S. africana* and the novel strain L21-Rpul-D2^T^ by a different salinity optimum. This classification scheme is also corroborated by the obtained cellular fatty acid data shown in the Additional file 2: Strain L21-Rpul-D2^T^ can be distinguished from all closely related type strains by the presence of the fatty acid ante-C_15:0_ and absence of C_18:1_c11, *S. africana* is characterized by the presence of C_16:1_c9 DMA and significant amounts of C_14:0_ DMA, whereas the unique feature of both species *S. asiatica* and *S. dissipatitropha* is the absence of the fatty acid iso-C_15:0_. The reclassification of *S. africana*, *S. asiatica* and *S. dissipatitropha* is however beyond the scope of this study and would require genome-sequencing of additional type strains. Formal descriptions of the proposed novel taxa follow below:

### Description of *Salinispira* gen. nov

*Salinispira* (Sa.li.ni.spi’ra. L. n. *salinum*, salt-cellar; L. fem. n. *spira*, coil, spire; N.L. fem. n. *salinispira*, a saline spiral)

Free-living, Gram-negative, slender and helical-shaped cells without hooks at ends and a width below 0.3 μm. Multicellular filaments are not observed. Rotation of cells and undulatory motility is conferred by two periplasmic flagella overlapping in the middle of the cell. No spores formed. Coccoid bodies resembling spheroplasts are formed in stationary phase cultures. Biomass has a slightly yellow-orange color due to carotenoid-like pigments. The diagnostic diamino acid of the peptidoglycan is L-ornithine. Major cellular fatty acids are C_14:0_, C_16:0_ and iso-C_15:0_. The polar lipid composition is dominated by phosphatidylglycerol, phospholipids, aminolipids, and glycolipids. No cytochromes or respiratory lipoquinones present. Tests for oxidase and catalase are negative. Aerotolerant, neutrophilic, mesophilic and moderately halophilic. Strictly fermentative metabolism. Amino acids or peptides are not utilized; thiosulfate or nitrate are not reduced. Yeast extract required for growth, but not vitamins. Resistance against rifampicin and kanamycin A. The type species belongs to the family *Spirochaetacea* within the order *Spirochaetales*.

### Description of *Salinispira pacifica* sp. nov.

*Salinispira pacifica* (pa.ci’fi.ca. L. fem. adj. *pacifica*, peaceful, referring to the Pacific Ocean, the origin of the type strain).

The main characteristics are as given for the genus. In addition, optimal conditions for growth are 35°C, pH 7.0 and a salinity of 50 g l^-1^ NaCl; salinities of up to 15% NaCl are tolerated. The following compounds are used for growth: N-acetylglucosamine, D-fructose, fumarate, D-glucose, D-maltose, D-mannose, pullulan, pyruvate, starch, and D-trehalose. No positive growth response is obtained with acetate, arabinose, butyrate, casamino acids, cellobiose, chitin, citrate, ethanol, formate, D-galactose, glycerol, lactate, lactose, malate, D-mannitol, melibiose, methanol, propionate, D-ribose, succinate, sucrose, D-xylose, and H_2_/CO_2_ (8:2 v/v). The end-products resulting from D-glucose fermentation are acetate, lactate, ethanol, CO_2_ and H_2_. Sulfate, sulfite, elemental sulfur, nitrate and nitrite were not used as terminal electron acceptors. In addition to the major fatty acids listed above, significant amounts of C_18:0_, ante-C_15:0_, C_18:1_, C_16:1_, iso-C_13:0_, C_16:0_ 2OH and iso-C_14:0_ are present. Susceptible to ampicillin (1000 mg l^-1^), carbenicillin (1000 mg l^-1^), penicillin G (1000 mg l^-1^), D-cycloserin (1000 mg l^-1^), chloramphenicol (20 mg l^-1^), gentamicin (1000 mg l^-1^), and tetracycline (500 mg l^-1^). The DNA G + C content of the type strain is 51.9 mol%.

The type strain is L21-RPul-D2^T^ (=DSM 27196^T^ =JCM 18663^T^), isolated from the suboxic zone of a hypersaline microbial mat at the shore of Lake 21, Kiritimati, Republic Kiribati.

## Competing interests

The authors declare that they have no competing interests.

## Authors’ contributions

SS and HPK developed the study concept. SS, MLF and HPK conceived and designed a majority of the experiments. WBH, MJ, and SS performed the experiments. AF, CS, PS, and BB contributed materials and analyses. All authors read and approved the final manuscript.

## Supplementary Material

Additional file 1 **Differential phenotypic characteristics of *Salinispira pacifica *strain L21-RPul-D2**^**T **^**and type strains of the phylogenetically closest related *Spirochaeta *species, as well as *S. halophila *and *S. smaragdinae.*** +, positive; -, negative; (+), weakly positive; *, results obtained in this study; ND, no data available. Strains and sources of data: *Salinispira pacifica* L21-RPul-D2^T^ (this study); *S. africana* Z-7692^T^[59]; *S. asiatica* Z-7591^T^[59]; *S. dissipatitropha* ASpC2^T^[60]; *S. halophila* RS-1^T^[61]; *S. smaragdinae* SEBR 4228^T^[62]. Click here for file

Additional file 2 **Cellular fatty acid patterns of *Salinispira pacifica *strain L21-RPul-D2**^**T **^**and phylogenetically related type strains of *Spirochaeta.*** Values are percentages of total fatty acids. Major fatty acids (>5% of total amount) are given in bold. Fatty acids that were detected only in trace amounts (<0.5% of the total amount) are not shown. ALDE, aldehyde; DMA, dimethyl acetal; c, *cis* isomer; iso and ante indicate iso- and anteiso-branched fatty acids, respectively. * Summed features are groups of fatty acids that could not be separated under the conditions used: summed feature 9, iso-C_16:0_ 3OH and/or unknown fatty acid DMA with ECL 17.157. Click here for file

Additional file 3 **Polar lipid pattern of strain L21-RPul-D2**^**T **^**revealed after two dimensional thin layer chromatography.** Staining of the chromatogram was done with molybdatophosphoric acid. Abbreviations: PG, phosphatidylglycerol; AL, unidentified aminolipid; PL, unidentified phospholipid; GL1 and GL2, unidentified glycolipids. Click here for file
