# Supplementary material for: Complete genome sequence and description of Salinispira pacifica gen. nov., sp. nov., a novel spirochaete isolated form a hypersaline microbial mat
Source: Stand Genomic Sci. 2015 Feb 9;10:7. doi: 10.1186/1944-3277-10-7 (PMC4511686; doi:10.1186/1944-3277-10-7)
Supplement: Additional file 1 — Differential phenotypic characteristics of Salinispira pacifica strain L21-RPul-D2T and type strains of the phylogenetically closest related Spirochaeta species, as well as S. halophila and S. smaragdinae. +, positive; -, negative; (+), weakly positive; *, results obtained in this study; ND, no data available. Strains and sources of data: Salinispira pacifica L21-RPul-D2T (this study); S. africana Z-7692T[59]; S. asiatica Z-7591T[59]; S. dissipatitropha ASpC2T[60]; S. halophila RS-1T[61]; S. smaragdinae SEBR 4228T[62]. [file 1944-3277-10-7-S1.docx]

**Additional file 1 – Differential phenotypic characteristics of *Salinispira pacifica*** **strain L21-RPul-D2^T^ and type strains of the phylogenetically closest related *Spirochaeta* species, as well as *S. halophila* and *S. smaragdinae*.**

| Characteristic | *Salinispira pacifica* | *S. africana* | *S. asiatica* | *S. dissipatitropha* | *S. halophila* | *S.* *smaragdinae* |
| --- | --- | --- | --- | --- | --- | --- |
| Cell width [µm] | 0.20-0.25 | 0.25-0.30 | 0.20-0.25 | 0.25-0.30* | 0.4 | 0.3-0.5 |
| Cell length [µm] | 8-9 | 15-30 | 15-22 | 8-18 | 15-30 | 5-30 |
| Pigmentation | + | + | - | +* | + | ND |
| DNA G + C content [mol%] | 51.9 | 57.8 | 49.2 | 43.8 | 62.0 | 50 |
| Temperature for growth [°C] |  |  |  |  |  |  |
| Range | 20-45 | 15-47 | 20-43 | 13-41 | 25-45 | 20-40 |
| Optimum | 35 | 30-37 | 33-37 | 35 | 35-40 | 37 |
| NaCl conc. for growth [g l^-1^] |  |  |  |  |  |  |
| Range | 20-150 | 30-100 | 20-80 | 10-30 | 3-73 | 10-100 |
| Optimum | 50 | 50-70 | 30-60 | 20 | 44 | 50 |
| pH for growth |  |  |  |  |  |  |
| Range | 6.5-8.4 | 8.1-10.7 | 7.9-9.7 | 7.8-10.5 | ND | 5.5-8.0 |
| Optimum | 6.9-7.0 | 8.8-9.8 | 8.4-9.4 | 10.0 | 7.5 | 7.0 |
| Aerotolerance | + | + | - | - | + | - |
| Oxygen respiration | - | - | - | - | + | - |
| Catalase | - | -* | -* | - | - | ND |
| Nitrate reduction | - | -* | -* | -* | + | ND |
| Vitamin requirement | - | + | + | + | ND | - |
| Yeast extract requirement | + | - | + | - | + | + |
| Utilization of: |  |  |  |  |  |  |
| Casamino acids | - | - | - | + | - | - |
| Citrate | - | -* | -* | + | ND | ND |
| Fumarate | + | -* | -* | -* | - | + |
| L-Lactate | - | -* | -* | - | - | - |
| Pyruvate | + | -* | -* | - | - | - |
| N-Acetylglucosamine | + | - | - | -* | ND | ND |
| D-Arabinose | - | - | (+) | + | + | - |
| Cellobiose | - | + | + | +* | + | ND |
| D-Fructose | + | + | - | + | + | + |
| D-Galactose | - | - | + | +* | + | + |
| D-Glucose | + | + | + | + | + | + |
| Lactose | - | + | - | - | + | ND |
| D-Mannose | + | + | + | - | + | + |
| D-Ribose | - | - | - | + | + | + |
| Sucrose | - | + | + | + | + | - |
| D-Trehalose | + | + | + | + | + | ND |
| D-Xylose | - | + | (+) | +* | + | + |
| Starch | + | + | + | + | + | ND |
| Glycerol | - | -* | -* | - | - | + |
| Ethanol | - | -* | -* | - | - | ND |
| D-Mannitol | - | - | + | + | - | + |
| Fermentation products |  |  |  |  |  |  |
| Hydrogen | + | + | - | + | + | + |
| Ethanol | + | + | + | + | + | + |
| Acetate | + | + | + | + | + | - |
| Lactate | + | + | + | +* | + | + |

+, positive; -, negative; (+), weakly positive; *, results obtained in this study; ND, no data available. Strains and sources of data: *Salinispira pacifica* L21-RPul-D2^T^ (this study); *S. africana* Z-7692^T^ [59]; *S. asiatica* Z-7591^T^ [59]; *S. dissipatitropha* ASpC2^T^ [60]; *S. halophila* RS-1^T^ [61]; *S. smaragdinae* SEBR 4228^T^ [62].
