# Supplementary material for: Complete genome sequence and description of Salinispira pacifica gen. nov., sp. nov., a novel spirochaete isolated form a hypersaline microbial mat
Source: Stand Genomic Sci. 2015 Feb 9;10:7. doi: 10.1186/1944-3277-10-7 (PMC4511686; doi:10.1186/1944-3277-10-7)
Supplement: Additional file 2 — Cellular fatty acid patterns of Salinispira pacifica strain L21-RPul-D2T and phylogenetically related type strains of Spirochaeta. Values are percentages of total fatty acids. Major fatty acids (>5% of total amount) are given in bold. Fatty acids that were detected only in trace amounts (<0.5% of the total amount) are not shown. ALDE, aldehyde; DMA, dimethyl acetal; c, cis isomer; iso and ante indicate iso- and anteiso-branched fatty acids, respectively. * Summed features are groups of fatty acids that could not be separated under the conditions used: summed feature 9, iso-C16:0 3OH and/or unknown fatty acid DMA with ECL 17.157. [file 1944-3277-10-7-S2.docx]

**Additional file 2 - Cellular fatty acid patterns of *Salinispira pacifica* strain L21-RPul-D2^T^ and phylogenetically related type strains of *Spirochaeta*.**

| Fatty acid | *Salinispira pacifica* L21-RPul-D2^T^ | *S. africana* DSM 8902^T^ | *S. asiatica* DSM 8901^T^ | *S. dissipatitropha* DSM 23605^T^ |
| --- | --- | --- | --- | --- |
| C_12:0_ | 0.8 | - | - | - |
| iso-C_13:0_ | 2.1 | - | - | - |
| iso-C_14:0_ | 1.6 | - | - | - |
| C_13:1_ c12 | - | 1.3 | - | **-** |
| C_14:0_ | **36.9** | **13.1** | **10.5** | **15.5** |
| C_14:0_ DMA | - | **5.9** | - | 0.9 |
| iso-C_15:0_ | **11.7** | 3.0 | - | - |
| ante-C_15:0_ | 4.3 | - | - | - |
| C_16:0_ ALDE | - | 2.8 | - | 4.2 |
| C_16:1_ c9 | 2.2 | **8.0** | **8.9** | **13.0** |
| C_16:1_ c11 | - | - | 2.3 | 4.3 |
| C_16:0_ | **21.4** | **19.9** | **37.6** | **21.2** |
| C_16:1_ c9 DMA | - | 0.6 | - | - |
| C_16:0_ DMA | - | **11.0** | 1.7 | **17.7** |
| ante-C_17:0_ | 0.6 | - | - | - |
| Summed feature 9* | 0.8 | - | - | - |
| C_16:0_ 2OH | 1.8 | - | - | - |
| C_18:1_ c9 | 2.8 | 0.9 | 0.9 | 0.5 |
| C_18:1_ c11 | - | **30.3** | **33.7** | **19.2** |
| C_18:1_ c13 | - | - | - | 0.5 |
| C_18:0_ | **9.7** | 0.8 | 3.2 | 2.0 |
| C_18:1_ c11 DMA | - | 0.8 | - | - |
| C_18:0_ DMA | 0.8 | - | - | - |
| C_19:1_ c7 | 0.7 | - | - | - |

Values are percentages of total fatty acids. Major fatty acids (>5% of total amount) are given in bold. Fatty acids that were detected only in trace amounts (<0.5% of the total amount) are not shown. ALDE, aldehyde; DMA, dimethyl acetal; c, *cis* isomer; iso and ante indicate iso- and anteiso-branched fatty acids, respectively. * Summed features are groups of fatty acids that could not be separated under the conditions used: summed feature 9, iso-C_16:0_ 3OH and/or unknown fatty acid DMA with ECL 17.157.
