# Supplementary figures and images for: Complete genome sequence and description of Salinispira pacifica gen. nov., sp. nov., a novel spirochaete isolated form a hypersaline microbial mat
Source: Stand Genomic Sci. 2015 Feb 9;10:7. doi: 10.1186/1944-3277-10-7 (PMC4511686; doi:10.1186/1944-3277-10-7)

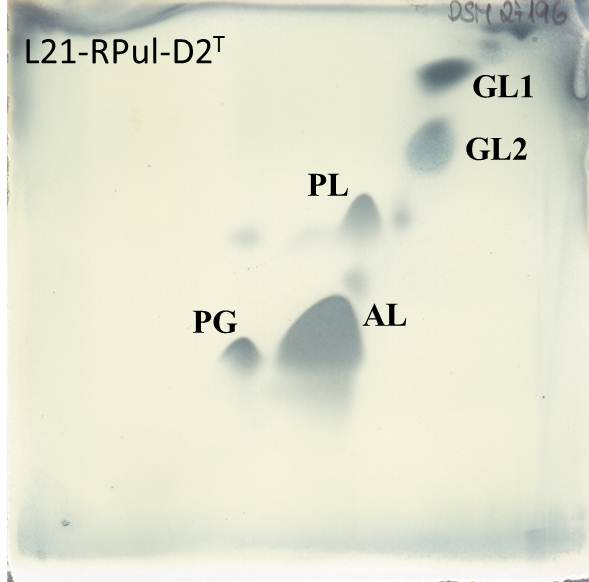

Supplement: Additional file 3 — Polar lipid pattern of strain L21-RPul-D2T revealed after two dimensional thin layer chromatography. Staining of the chromatogram was done with molybdatophosphoric acid. Abbreviations: PG, phosphatidylglycerol; AL, unidentified aminolipid; PL, unidentified phospholipid; GL1 and GL2, unidentified glycolipids. [file 1944-3277-10-7-S3.jpeg]
